# “They Care a Lot”: A Qualitative Study of Nurses' Perspective on the Performance Appraisal Process in Israel

**DOI:** 10.1155/jonm/5525447

**Published:** 2025-09-30

**Authors:** Edna Rabenu, Yonatan Shertzer

**Affiliations:** ^1^Human Services Department, Tel-Hai College, Upper Galilee, Tel-Hai, Israel; ^2^Human Resources Department, Peres Academic Center, Rehovot, Israel

**Keywords:** nurses, nursing management, performance appraisal, performance evaluation, thematic analysis

## Abstract

**Intro:**

The performance appraisal (PA) process is carried out in organizations in general and in health organizations in particular due to its expected benefits, such as improving performance regarding patient care quality, documentation, identifying training needs, raising job satisfaction, and employee engagement. This paper explores the nurses' perspective on the PA process.

**Design/Methods:**

As part of course assignments in the Master's degree program in health systems management, 34 nurses in hospitals in Israel (students in the course) were asked to describe the PA process they underwent in their organization (their written narratives regarding the PA process). Thematic analysis was employed to examine their responses.

**Results:**

Four themes were found: characteristics of the feedback session (the PA interview), characteristics of the appraisee, characteristics of the appraiser, and characteristics of the PA process.

**Conclusion:**

Beyond elucidating the nurses' subjective experiences and priorities, the study brought to light several major issues, such as the fact that nurses highly value the PA process and do not consider it a mere formal chore. Also, the nurses emphasized the procedural justice of the process (i.e., they cared that the process has sufficient validity and reliability). Lastly, they crave recognition and acknowledgment.

**Implications:**

This research has theoretical and practical implications based on specifying the nurses' perspectives and priorities and taking them into consideration. The study's findings can assist healthcare organizations to understand this crucial practice better so that it can achieve its full potential.

## 1. Introduction

Nurses are the backbone of the health system and are essential in ensuring that patients receive the care they require [1, 2]. As such, appraising nurses' performance is essential for upholding high standards of care, pinpointing areas in need of development, and encouraging career advancement [3].

Performance appraisal (PA) can be defined as “…a formal, well-planned organizational process designed to obtain reliable and precise information about the job performance of a certain employee and his/her job-related behavior” [4]. Accordingly, PA is a tool that aids in identifying and differentiating between exceptional, average, or inadequate employees based on criteria that are critical in determining satisfaction from their job performance [5]. For example, according to Kahya and Oral [6] research based on clinical nurses in hospital, the most prevalent categories for PA are “clinical skill” (e.g., planning patient care according to individual needs and managing the nursing activities in time), “professional skill” (e.g., identifying and assessing of the patient's problems; keeping nursing equipment in good condition), and “contextual performance” (e.g., working hard with extra effort; working systematically). The other five categories of PA criteria include interpersonal skill, problem solving, professional ethic, teamwork, and leadership.

To illustrate, based on the teamwork criteria of PA, outstanding nurses will actively participate in team meetings, collaborate across departments, give constructive feedback to colleagues, and contribute to evidence-based practices, while inadequate nurses might avoid team discussions, give vague or too critical feedback, and show little involvement in research-based initiatives of the team [6]; for further criteria for nurses PA, see Jerónimo and Araújo [7].

The PA process entails compiling information from diverse sources, such as firsthand observation, patient comments, peer assessments, and self-evaluations. After that, these data are examined to determine the nurses' strengths, shortcomings, and possible training requirements [8].

Several advantages can be obtained from efficient PA systems in the nursing profession. Firstly, nurses can improve their skills and knowledge, which improves patient care and clinical outcomes, by recognizing and addressing performance gaps [9]. Secondly, nurses can use the insightful feedback from performance reviews to direct their efforts toward professional development, pinpoint areas in need of additional training, and explore opportunities for career advancement and talent management [3, 7]. Thirdly, performance reviews encourage transparency and accountability making sure adhering to a set of standards of practice and support the upkeep of a top-notch healthcare system [10]. Fourthly, it can improve communication between the appraiser and the appraisee. It is a formal moment for sharing and discussion between both parties [7]. Fifthly, the PA process can strengthen job satisfaction and motivation: Nurses are more likely to be engaged and productive in the workplace when they receive regular feedback and acknowledgment for their achievements [11, 12]. And lastly, nurses who receive performance evaluations are more likely to work toward a culture of continuous improvement, which motivates them to pursue excellence and instills a sense of shared accountability for patient care [13]. Indeed, a recent systematic review examined the impact of annual PA processes (focusing on constructive feedback, transparency and fairness, and professional development) on job performance and satisfaction among nurses, analyzing 11 cross-sectional studies (selected from 764 primary publications in English between 2012 and 2022) which reveals that structured PA processes, which include constructive feedback, fairness, transparency, and clear definition of expectations, contribute to improving nurses' performance and increasing their job satisfaction [14].

Despite the PA process's many benefits, there is substantial disagreement regarding its efficacy [15, 16] and its suitability to the contemporary work environment [17, 18]. Murphy [19] suggested that there are four main shortcomings of the PA process: (1) Most PA processes operate under the assumption that performance is normally distributed, but this assumption has been proven wrong in many studies [20, 21]. (2) Evaluations of employees' work performance are almost invariably based on the subjective assessments of peers, supervisors, or other sources [15]. Therefore, employees often perceive the PA process as unfair, biased, and political [22]. (3) There is limited value of performance reviews for staff members. Correspondingly, in their comprehensive review of research on the impact of feedback, Kluger and DeNisi [23] found that feedback improved performance in only about one-third of the studies they examined, and that about the same proportion of studies showed that receiving feedback results in lower performance. (4) PA process has limited value to organizations. Several studies questioned the effectiveness of achieving its goals [15, 24].

In accordance with the shortcomings presented by Murphy [19] (excluding reference to the first one), Jerónimo and Araújo [7] found that the PA process of nursing suffers from several fundamental shortcomings: It is a complex and bureaucratic process with lack of sufficient preparation for using the system. It is inaccurate and is not supported by the reward system. Also, it does not contribute to professional advancement. All these shortcomings create a sense of obligation and frustration among the appraised nurses [7]. Indeed, Jaber and colleagues [25] highlight the considerable influence that poorly designed PA systems, ineffective tools, and inadequately trained appraisers can have on organizational commitment, job satisfaction, the retention of a dedicated nursing workforce, and the overall quality of healthcare services. Moreover, the study underscores the critical importance of implementing robust and effective PA systems within healthcare organizations.

Based on the described above shortcomings in the PA process and following the recommendations of Jerónimo and Araújo [7] for the need to deepen the reflection and develop new research in this field of PA of nurses, we would like to examine the perceptions of nurses regarding the PA process, as well as their perspectives and interpretations of it; the nurses' point of view as appraisees (being evaluated) on the PA process can lead to further improvements in the process and minimizing its obstacles.

Social exchange theory (SET) provides a useful perspective through which to examine performance evaluations in general [26–28] and nursing in particular. According to this theory, workplace relationships are based on reciprocal exchanges of resources, both tangible and intangible, where individuals weigh costs against benefits [29]. In the context of performance reviews, nurses and their organizations and managers have a complex exchange relationship in which the nurses' professional contributions, commitment, and quality of care are traded for acknowledgment, career development, and equitable evaluation by managers. The theory explains how nurses' perceptions of evaluation fairness and reciprocity can significantly impact their job satisfaction, organizational commitment, and subsequent performance. When nurses perceive their performance evaluations as balanced and just, they are more likely to invest their resources in their roles, leading to improved patient care outcomes and organizational effectiveness [26, 30]. Moreover, Jaber and colleagues [25] key insight from the research was the pivotal role of mutual understanding between the appraiser and the appraisee (nurse being evaluated); this relational dynamic significantly enhances the effectiveness of the appraisal process and reinforces its value as a developmental tool.

Therefore, the study's goal is to investigate how nursing staff members feel about the organization's PA procedure, what their emphasis was when describing the process, and to understand the emotional and practical expectations they have from the process. By understanding the process from the nurses' point of view, health institutes will be able to generate practices and policies that can assist the appraisers of the process (e.g., head nurses) to improve performance, job satisfaction, organizational commitment, and hopefully minimize negative phenomena such as stress and burnout that nurses experience in their role [31, 32].

## 2. Materials and Method

In the current study, we aim to gain a thorough understanding of the phenomenon of PA and the experience and meaning that nurses attach to it. The purpose of the study is to examine the stories of nursing workers regarding the PA process they went through in the organization. The research is exploratory, without preconceived notions, with the aim of openly exploring the phenomenon.

The research was conducted as part of course assignments in the Master's degree program in health systems management. One of the authors personally referred to the students to complete this assignment as part of their academic course. The students were all healthcare employees, including nurses, and they completed the assignment about their own work. The only question the subjects were asked to answer was “Tell me about the PA process you went through in the organization?” following by further several background questions. The full assignment is attached in Appendix A.

The students were given this assignment in the first lesson of the semester and within a very limited time frame, while their knowledge in the field of PA was still quite rudimentary. The goal was to document their stories before they were influenced by study material that might create social desirability (for instance, reporting what they should do according to academic literature). Additionally, the students were explicitly told to provide raw material, namely, to present their personal stories regarding their PA process without scholarly and educated interpretative analysis. They were clarified that there is no right or wrong answer. The students chose what to disclose (e.g., they were allowed to describe the technical procedure without emotional references or to express their emotions concerning the process they underwent).

It was explicitly stated that if they write at least half a page unfolding their story of PA from their perspective, they will have successfully completed the task. This way, there was no pressure on the students to express specific ideas in their stories.

Before starting working on the assignment, all participants received a detailed and clear group explanation via Zoom about the goals of the study, the nature of the assignment, the length of time required, and the expected benefits (no risk was expected). It was explained that participation in the study was completely voluntary, and it was clarified that each participant had the right to withdraw from the study at any stage and without any consequences or need for explanation. Also, they were told that the mere act of writing a story and submitting it to a protected electronic assignment box was enough to pass the assignment (pass/fail grade) regardless of the content of the story. By filling out the background questions, they gave their consent to participate in the research. It is important to note that the students could also refuse and declare their noninterest in their data being used for research purposes. It was stated and written explicitly.

This course assignment (pass/fail grade) was 5% of the total grade. The grading for the stories was given at the beginning of the course. All students in the course passed this assignment, regardless of its quality. The disadvantage of using the assignment was that there was a very slight inflation of the grades in the course (everyone passed the assignment and received five points), but we believe it prevented further potential problems for the research such as writing stories in a specific way only because the lecturer in the course has power over the students and/or they want to please the lecturer under social desirability issues. There were a few students on the course who, fortunately, were not nurses (but served in other roles in health care) and therefore not included in the research. All the nurses chose to participate; however, four nurses who submitted the assignment and passed it were not included in the research because their assignment did not answer the question of the PA story:

Nurse J (hospital) did not tell a story but copied the PA form she received.

Nurse L (HMO) talked about being a nurse in a hospital in the Soviet Union and the challenges of her professional transition.

Nurse I (hospital) wrote about a problem she encountered with the computer system at work and her need in guidance with the computer system.

Nurse H (hospital) had only just started her nursing career and had not yet passed a PA.

We would like to point out that most of the students wrote much more than the minimum required to complete and pass the task. After the grades were distributed (all passed the assignment), the researcher who gave the task asked the class why (in general) they wrote much more than required, and they noted that finally someone asked them about their PA process and that they had a lot to share on the subject. After receiving the grade, students could also request that their stories not be used for research purposes without any negative consequences (as the grade had already been given). Furthermore, the students were informed from the start of the course that the final exam would be anonymous (and multiple questions), so as not to create bias in our evaluation (based on my impressions from the narrative stories) during its assessment.

For research purposes, only nurses who wrote about PA process they went through in their organization were entered as participants. As such, the participants are nurses in the healthcare system across Israel (There are currently approximately 85 hospitals in Israel, encompassing a range of types including general hospitals, psychiatric institutions, geriatric centers, and private medical facilities. The hospitals operate under various ownership structures: governmental, public, and private). A total of 32 of them are nurses in hospitals in variety of departments, one is in HMO, and one in public health. As far as we know, there are no participating nurses who share both the same hospital and the same department, so the representation is quite diverse (see Table 1).

The use of the PA stories was made anonymously using codes instead of names, without personal details that could identify the students. The students' stories in their entirety, including the full background data, were kept on the first author's personal computer, to which she only had an access code. Verbatim quotations are used to illustrate participants' experiences and have been retained in their original form to preserve authenticity. The study was approved by an institutional ethics committee on Netanya academic college (number 2/2021), while ensuring that the ethics rights of the participants will be fulfilled.

We analyzed the interview transcripts using inductive thematic analysis to identify and interpret recurring patterns across participants' accounts. Coding began without a predefined framework and was developed directly from the data. An iterative process of comparison across transcripts allowed us to refine and consolidate codes into broader themes that captured the central aspects of participants' experiences. This approach ensured that the analysis remained grounded in the data while systematically progressing from initial codes to higher-level thematic insights [33, 34].

We employed theoretical sampling—we chose an initial sample of 10 nurses to construct categories and then tested them against additional samples (another 10 and then another 14 nurses) until we reached theoretical saturation [35]. The nurses' details (gender, age, seniority, and type of institution/department) are presented in Table 1.

Our form of sampling is maximum variation sampling, which purposively selects a wide range of cases [36]—nurses from hospitals, community clinics, old people's homes, family health stations, and “Tipat Halav” (stations that provide health and medical services in the field of health promotion and prevention for pregnant women, infants, and children). This qualitative approach of maximum variation sampling brings the personal experience of each nursing worker from the PA process and at the same time creates a meaningful integrity in the understanding of the process for other nursing workers.

## 3. Results and Discussion

### 3.1. Results

We found four themes: characteristics of the feedback session (the PA interview), characteristics of the appraisee, characteristics of the appraiser, and characteristics of the PA process. See Table 2.

#### 3.1.1. Characteristics of the Feedback Session (the PA Interview)

##### 3.1.1.1. The Location

A considerable number of nurses opted to include the feedback session's location in their narrative. Mostly, the session was held behind closed doors in the head nurse's office. For example, nurse 1F: “*The door of the room was shut … Her desk was free of binders and pages except for my evaluation form*”.

##### 3.1.1.2. Emotion Management in the Session

Some of the nurses emphasized the fact that the situation of the feedback session is stressful and having a conversation that “defrosted” the atmosphere and manage the stress they felt in the session, rather than just talking on performance issues was important to them (such asking how they were feeling, their overall feelings in the department, and how they were handling the work). For example, nurse 1I said “*The first thing that my supervisor asked me is how I was feeling and how my kids were. From that moment I felt relief, I understood that I worked in an organization that treats me not only as an employee but also as a woman and a mother*”. Another example is nurse 3E: “*The session opened with how I was doing, my feelings in the department, and how was I connecting with my colleagues*”. Last example is nurse 1A: “*The high level of stress remained, despite the many evaluations I went through, with the current nurse in charge as well as with her predecessor. I remember the excitement, the blush on my cheeks and the dryness on my lips. At the same time, I always tried to maintain restraint and look cool and listen to everything she said*.”

##### 3.1.1.3. Improving the Appraisee Performance

Predictably, most of the session focused on enhancing the nurses' performance. This includes the following methods:

###### 3.1.1.3.1. Setting Goals

A few of the nurses were given certain objectives to fulfill in the upcoming year. This could include training, reaching a professional level, and enhancing certain areas of their work. For example, nurse 3D said “*We discussed the objectives I must meet. Enrolling on a shift manager course was one of my objectives, as it would enable me to cover for the department head during his absences*”.

###### 3.1.1.3.2. Concerns for Improvement or Preservation

A few of the nurses reported that the appraiser had talked to them about their strengths and some areas where they needed to improve. For example, nurse 1J said “*The head nurse made note of my areas for improvement as well as what sets me apart from the rest of the group, namely, my accountability and my punctuality*”.

###### 3.1.1.3.3. Offering Guidance and Assistance

A few of the appraisers inquired about the appraisee's needs and gave them advice on any problems they were having. For example, nurse 2I said “*She mentioned that I can approach her with everything I need or any weaknesses I require her help with*”. Also, nurse 1F said “*My manager offered to guide me and help me improve certain aspects of my work for the upcoming period*”.

##### 3.1.1.4. Career Development

Nearly every nurse mentioned that the session referred to their career as a nurse. Some of the nurses mentioned some courses they were offered the chance to go to or asked on their own initiative to attend in the previous year. For example, nurse 2E: “*We talked about the palliative care course I took … and agreed that the next stage was an intensive care course*”. A few nurses discussed their plans for the future of their careers, including their aspirations and whether they wanted to be promoted to higher positions. For instance, nurse 1J: “*The first question I was asked is where I saw myself in 10 years' time. This made me think whether I wanted to be a certified nurse or pursue a more managerial role*”. Several nurses stated that the appraiser encouraged them, believed in them, and that they could advance in their career. For instance, nurse 2J: “*In every session she told me that I could go very far in this vocation, and that she always thought highly of me*”. Finally, some of the nurses reflected on the effect that the performance evaluation had on their career. For instance, nurse IG: “*I received some feedback and evaluation throughout my career, especially when I started, that had a major role in my professional development*”.

##### 3.1.1.5. Bottom-Up Feedback

A few of the appraisers inquired about any feedback the nurses would like to provide in their capacity as head nurses or department heads or inquired about their professional opinions regarding the state of affairs in the department. For example, nurse 3N said “*The head nurse asked me if I had any opinions about the staff or her. I made some suggestions regarding the department we collaborate with and about a few of the recently hired nurses. I also offered some recommendations for how to enhance our department's operations*”.

#### 3.1.2. Characteristics of the Appraisee

##### 3.1.2.1. Tenure

The tenure of the appraisee (in the organization or generally in the profession) at the time of the evaluation is a crucial component of the appraisal process. We will discuss this topic later but let us state up front that the nurse's tenure has a significant impact on the overall attitude toward the evaluation and the appraisee's openness to feedback.

###### 3.1.2.1.1. Tenure in the Organization

Some of the nurses emphasized their tenure in the organization as part of their professional identity or credentials as a nurse. For instance, nurse 1A: “*Despite being a ‘veteran' nurse for almost 14 years in the department…*” or nurse B1 “*I have been working as a registered nurse in the department for about 13 years*”.

###### 3.1.2.1.2. Tenure in the Profession

Some of the nurses were perhaps new in the department or the organization, but they had a lot of nursing experience from previous places. Again, as in tenure in the organization, the nurses emphasized that they were experienced, especially if they were new to the department. In some cases, the nurses alluded to the fact that even though they were experienced as nurses, their new job was novel to them. For example, nurse 1I: “*I went from working in a hospital to working in the community as part of ‘Tipat Halav'. I was accepted to work at the family health station by a team of nurses who are very professional in their field and an instructor nurse who put me to work, after almost 13 years in a hospital as a nurse,… I felt like I was starting all over again; the field is very new to me*”.

##### 3.1.2.2. Attitude Toward the PA Process

We have noticed considerable differences in the attitude of the nurses toward the process. We have marked each interview with the nurses' general tone and labeled each one as belonging to one of three categories: positive, negative, or neutral.

Positive tones are characterized by feelings of warmth, care, and as an opportunity for growth. For example, nurse 1G: “*I felt comfortable in the conversation and talked with great confidence because I felt he cared about me and every word I said counted*”. Another example is nurse 3G: “*Even when the evaluation had a lot of critique about me, I understand that the ultimate goal was to serve as constructive feedback, and I use it to recognize my weaknesses or improve my performance*”.

Negative tones exemplified feelings of unfairness, cynicism, and a feeling that this process had little to benefit from. For example, nurse 3C: “*I have nothing good to say, such frustration, I feel that the process was done casually and only to mark V*”. Or nurse 2F: “*My experience from the evaluation appraisal hasn't really changed in the last 4 years, I have the same manager who provides a laconic appraisal. She hates the process, and I can sense that for her it's just another chore, and I know now that this won't change until she leaves*”.

Neutral tones were one of two things. Some of the nurses described the process very technically and did not express any emotion about it. Other nurses were ambivalent about it and mentioned positive and negative aspects of it. For example, nurse 1D “*I am ambivalent about the process. On the one hand I have an open and honest relationship with my manager, and I usually get favorable appraisals. On the other hand, in the 5 years I have worked here I haven't done anything that was new to me, and therefore I believe that specifically for me the process is redundant*”. We have noticed that most of the negative tones belonged to more experienced nurses who underwent this process numerous times and perhaps feel the process had little to offer them.

##### 3.1.2.3. Need for Appreciation

On top of being open to feedback, the PA included an important aspect of need for appreciation and respect. For instance, nurse 1G: “*I had great satisfaction … I felt joy and self-actualization and the feeling that I led a project, and someone saw that and appreciated my hard work*”. Also, nurse 2J: “*I always liked it when the head nurse called me in for the evaluation because I love to be complimented on a job well done, and on my personality*”.

Unfortunately, some of the nurses felt that the PA did not satisfy their need for appreciation. For example, nurse 3C: “*I always felt that something was missing … that the evaluation process was another chore for the head nurse … I feel like I am giving my heart and soul, and the moment goes by in a blink of an eye without talking about my feelings or complementing me about my performance*.”

##### 3.1.2.4. Personal Context

The PA does not occur in a vacuum. Some nurses have mentioned the timing of the situation and background regarding their personal life. For example, nurse 2J: “*I remember one time she gave me a coupon for breakfast because I left my 2 years old son with a high fever and came to work to cover for other nurses who were on retreat*”. Another example is nurse 1G: “*Last year I gave birth to my third child, usually the time after childbirth is difficult. I needed to get accustomed to the new reality with three children at home and on top that, four months later the COVID-19 pandemic started … The head nurse called and said I needed to make time for a Zoom conversation so I could receive the PA … Naturally I made myself available and tried to find time when I wouldn't be disturbed … The head nurse asked me for feedback on every aspect of the assessment and advised me on how to improve my work performance, which is of utmost importance … What I recall the most is that she told me that I was an exemplary employee*. *This was so moving for me in that context on the maternity leave when I felt so inactive*”.

#### 3.1.3. Characteristics of the Appraiser

##### 3.1.3.1. Appraiser's Professionalism Regarding the PA

###### 3.1.3.1.1. Strictness

Some of the nurses related to the head nurse's strictness and adherence to the PA form, which manifested itself in how meticulous she or he was when they gave the PA. For instance, nurse 1G: “*The head nurse went into great detail with me regarding every clause in the PA. For each grade she gave me, she provided me with actual examples. She also stressed what I could do to improve what needed to be improved, and asked me what I thought of everything she had told me*”.

###### 3.1.3.1.2. Seriousness

Many nurses thought the head nurse took this *process* seriously, and they also thought the preparation on her part was important. Of course, there was a wide range here, from head nurses who seemed uninterested in the process to those who gave it a lot of consideration; for example, nurse 3A said “*The head nurse came prepared with a case study, she collected all the data concerning this case study, double checked them, and came prepared with a presentation on the subject. She could make an exact estimation of my contribution to the department and the entire organization at any given time, even on the night shifts*”. On the other hand, we can see an example of a head nurse who did not take the process seriously. Nurse 2F: “*I felt like she didn't bother to learn what I did and how I really performed*”.

##### 3.1.3.2. Motivation to Appraise

Some of the nurses mentioned whether the appraisers had any motivation to give them the PA. For example, nurse 2A: “*The evaluation was quite laconic. The head nurse doesn't like to give evaluations and she gives the feeling that this is a burdensome chore for her. I understand that this won't* change *until she leaves*”.

##### 3.1.3.3. Expressing Emotions

A recurring theme in the description of the evaluations was whether the appraisers were warm or displayed emotions toward the nurses. This seems to be a very essential aspect of the evaluation on the nurses' part. Some of the nurses mentioned physical contact such as a hug, and in general, the head nurses were kind. For example, nurse C2 describes the behavior of the appraiser as “very warmhearted” and nurse 2J said “*She gave me a total of three evaluations, each of them ended with a hug from her*”. Another example is nurse 1A: “*I felt comfortable in front of my supervisor as if she was my mother who came to talk to me*”.

#### 3.1.4. Characteristics of the PA Process

This last part concerns issues of the PA process in general (not the specific session).

##### 3.1.4.1. Frequency and Timing

Most nurses stated that they undergo an annual evaluation; however, a small percentage also stated that they receive one evaluation every 2 years or less, twice a year, or even every quarter (only one nurse reported a quarterly evaluation).

A few nurses expressed their dissatisfaction with the matter, claiming that an annual performance review is insufficient. They recommended that they should be assessed more regularly, and that this could contribute to their performance at work. Nurse 3C, for example, stated “*The process's format needs to be updated to better reflect modern times. We ought to conduct assessments more frequently and receive short-term objectives. We occasionally receive training, but there is no oversight of how we put what we learn into practice*”. Since most evaluations are conducted once a year, many nurses reported that PA occurred at the end of the year or early in the following year. In uncommon circumstances when the PA was conducted twice a year or quarterly, the session was held at the conclusion of the time frame for which they were assessed (end of the quarter or end of the half of the year).

##### 3.1.4.2. Justice

The PA process is typically linked to numerous organizational decisions or processes, such as bonuses, layoffs, promotions, and salary increases. Given the importance of the process in making various decisions, and the reasonable belief that it will significantly affect people's lives, it is not surprising that appraisees place high value on the process' perceived fairness.

###### 3.1.4.2.1. Procedural Justice

The definition of procedural justice is the fairness of the procedures leading to results. In the case of PA, it relates to the perceived validity and reliability of the process. Some of the nurses questioned whether the manager could accurately give them feedback. For example, nurse 3H: “*My manager can't fairly criticize me about how I perform because we work in shifts. She relies solely on the unfounded opinions of other nurses, and never really saw me working at night*”. Also, nurse 3M said “*I have many roles but since they are not evaluated separately, I have nothing to do with the criticism I received*”.

On the other hand, when nurses believed that the PA was based on reliable or valid sources, they readily agreed with the appraisal. For instance, nurse 3J: “*The PA is based on feedback from patients and their families, other team members, and of course on how well I perform*”. Another example is nurse I3: “*I felt that the head nurse really follows our performance and doesn't rely on rumors. She evaluates us objectively and it made me want to be just like her*”.

Additionally, some of the nurses mentioned the importance of relying on multiple sources of information, to have a more comprehensive perception of their performance; for example, nurse 2A said “*The head nurse says she doesn't know me, but she receives frequent reports from the shift manager, other nurses, and, of course, the mothers who I assist with breastfeeding*”.

###### 3.1.4.2.2. Interactional Justice

  Interpersonal Justice. The nurses described how well the appraisers carry out procedures or make decisions, while treating the appraisees with decency, respect, and civility. This type of justice was mentioned by many of the nurses. For instance, nurse 2B: “*From the start she [the head nurse] gave me the opportunity to share my feelings, express myself, tell her whether I was satisfied with my work or not, and if there were additional things I wanted to tell her*”. Or nurse 1J: “*I felt comfortable and spoke with confidence because I felt he cared about me and took me seriously*”.
  Informational Justice. Informational justice is concerned with the justifications given to individuals explaining why certain procedures were followed or results were given. The perceived level of informational justice is higher in areas where explanations are more adequate. Specifically, some of the nurses felt that the way the head nurse explained the PA was unfair. Some of them mentioned that, at the end of the conversation, they had to sign whether they agreed or disagreed with the feedback. A few nurses mentioned they disagreed or reluctantly said they agreed to sign, even though they thought it was unjust. For example, nurse 2F mentioned that “*In hindsight, the evaluation didn't reflect reality … I felt she didn't know me and what exactly my roles were … I regret signing that I agreed with the evaluation … I hate to say it, but what she wrote doesn't reflect reality and many of the things she wrote are quite different*”. Another example is nurse C3: “*Usually our supervisor fills out the assessment alone and, at the event, she reads only the last page and lets us add comments or clarifications, and sign*”.

##### 3.1.4.3. Goals of the Process

Most nurses shared their view about what they thought the goal of process was. We can see there is a great variety of perceived goals.

###### 3.1.4.3.1. Management of Career Paths

The PA process is used as a criterion to monitor the placement and development system, while providing information for decision-making: (1) Is the employee suitable for this position? and (2) Does the employee have potential for promotion? For example, nurse 1A: “*The goal of the process is to get certain insights about personal decisions I need to take about my job*”. Also, nurse 1E said “*This is a great opportunity to see if I and the head nurse are on the ‘same page'. This will help me to understand how to realize my potential*”.

###### 3.1.4.3.2. Professional Guidance

Some nurses mentioned that the goal of the process is to guide them on how they should develop professionally. For example, nurse 1D: “*It is important that I know what the head nurse thinks of me, and understand if there are some areas in my work I need to improve*”. Nurse 3B added *“The process goal is to be an organized procedure, to set personal and professional goals, and to improve us as nurses*”.

###### 3.1.4.3.3. Open Communication

Some nurses believe that the PA process provided them with a rare opportunity to be heard by their superiors and express themselves. Nurse 1H: “*The PA* that is *held annually in a laidback atmosphere gives me the opportunity to express how I feel*”.

###### 3.1.4.3.4. Improvement of the Department

Some nurses stated that the process goal is continuous improvement not only of themselves but the entire department or even the organization. Nurse 2E: “*The process is important for us to constantly improve the quality of our work and the nurses' job satisfaction*”.

##### 3.1.4.4. Criteria for Evaluation

The nurses were evaluated on various elements. In the following section, we discuss these various elements:

###### 3.1.4.4.1. General Assessment

Some nurses mentioned that the evaluation included a component that related to the general evaluation by the appraiser**—**whether beyond all specific issues they were content with the nurse's performance. For instance, nurse 1E: “*The session ended with a general assessment of my performance concerning whether I succeeded in my job or not*”.

###### 3.1.4.4.2. Contribution to the Organization's Success

A portion of the PA dealt with the role that nurses played in the organization and its success. This was mentioned in several places, including helping the organization achieve its goals, being committed to and identifying with its values, and actively contributing to the success of the department and the organization. For instance, nurse 1B: “*I was evaluated on how I promote achieving the organization's goals*”.

###### 3.1.4.4.3. Direct performance

Naturally, the evaluation revolved chiefly around the nurses' work performance. This part consisted of three main criteria: performing the tasks, meeting goals, and the manner in which the tasks were carried out.

  Performing the Tasks. The nurses were evaluated about performing their various tasks and chores in the hospital. For example, nurse 1E: “*I was evaluated about various chores I perform;* for example*, taking holistic care of the patient, working in compliance with hospital rules and the Ministry of Health regulations, creating a therapeutic connection with the family … ensuring conditions for maintaining the patient's safety in providing drug treatment and preventing infections, identifying life threatening situations, and providing the best possible treatment within the boundaries of my authority*”.
  Meeting Objectives. Some of the nurses stated that the PA included an assessment of whether they met or did not meet their periodic goals. For instance, nurse 1F: “*The head nurse evaluated whether I managed to successfully complete the objectives she set for me: building a new training system for new employees with emphasis on infection prevention, carrying out four trainings for the nursing staff during team meetings that included a lecture presentation and a knowledge test, implementation of covert observations with hand hygiene on the teams, and raising compliance with hand hygiene to 80% compliance*”.

###### 3.1.4.4.4. Indirect Performance

The PA processes also refer to indirect performance, which takes into account how the appraisees did their tasks and not only if the tasks were completed. The nurses were evaluated about the manner they operated in their various tasks. Two role-related behaviors that were particularly emphasized could be observed:

  Initiative Behavior. The nurses were assessed for behaviors of initiative and action beyond what was required in their role. For example, nurse 1E: “*…I was also evaluated for initiative and innovation;* for example*, promoting improvement and innovation in areas of my responsibility, identifying future needs, showing flexibility in dealing with unplanned tasks, showing willingness to perform additional tasks when required, and promoting knowledge expertise in areas of responsibility. Lastly, I was evaluated about my work values*—*identification and commitment, being a role model … attending work as required, being a positive person …*”
  Relationship With Other People. Since being a nurse involves working with other people, this subject was mentioned by most nurses as a criterion they were evaluated on. The nurses were evaluated about working with patients, work interfaces, and working with the other nurses and their superiors. For instance, nurse 2E: “*I was told that I support the employees, especially the new ones, that I aid in practically every shift, that I maintain a polite and respectful demeanor with the staff, and that I am available to help in an emergency. I was informed that my supervisor was appreciative of my help with CPR for the other departments, and did not take it for granted. I was also told that I foster a positive environment with the patients and the team*”.

###### 3.1.4.4.5. Professional Knowledge

This issue may fall under performance, but we felt it was important enough to recognize it as a distinct issue given its relatively significant presence. Many nurses reported that their professional knowledge was evaluated, and it appears that this is a fundamental requirement in this line of work. For example, nurse 3G: “*Among other issues, I was evaluated on my clinical knowledge and its implementation in my everyday work*”.

## 4. Discussion

This study provides a distinctive viewpoint on the key mechanisms acting on nurses' PA process. The qualitative analysis has shown the main issues that nurses attach to PA. As we have shown, the results are divided into four themes: characteristics of the feedback session (the PA interview), characteristics of the appraisee, characteristics of the appraiser, and characteristics of the PA process. We would like to draw attention to a few points that our analysis raised.

The first point is the nurses' perceived importance of this supposedly administrative procedure. It is evident that the nurses regarded the process as an essential tool for their professional and personal development, wrote about it with great emotion, and took it very seriously. Most (if not all) of the nurses agreed on the process' significance, despite their differences in how they felt about accepting the feedback. This finding corresponds well with the literature that indicates despite various claims that are made against the PA process: its low frequency, its biases, its generality as opposed to the personalization of the process, and so on; see, for example, [17, 19]; its advantages still outweigh its disadvantages. Employees wait for a process in which they will be evaluated for their performance, even if they fear or are frustrated by it for various reasons [4, 18].

The second point we would like to address concerns the issue of justice. Although the nurses were only asked about the PA process, without any reference to matters of justice, they perceived the issues of justice as a critical part of the process. This finding supports previous research on nurses (e.g., [14]). Thurston and McNall [22] developed 10 scales to provide an empirical evaluation of the underlying dimensions of organizational justice in PA (assigning raters, setting criteria, seeking appeals, equity decision norm, absence of political goals, respect in supervision, sensitivity in supervision, clarifying expectations, providing feedback, and explaining decisions). As can be seen in Table 3, our findings have good match between Thurston and McNall's [22] 10 scales and with the dimensions of organizational justice related to them. However, there are three major differences:

1. Thurston and McNall [22] did not address the “characteristics of appraisee” theme, whereas in our study this theme occupies an important space. We believe that this theme is a central contribution to this study as it reinforces the fact that in PA process, we must remember that “it takes two to tango”, especially when considering the PA process from a relationship and social exchange perspective. The SET addresses that social relationships are based on the trust that gestures of goodwill will be reciprocated [29]. Previous studies already regarded justice dimensions as part of exchange in workplace. For example, a meta-analysis found that variables such as trust, perceived organizational support (POS), and leader–member exchange (LMX) were important to relations among task performance, justice, and citizenship behavior [38]. In the same vein, recent studies by Uraon and Kumarasamy [27, 28] demonstrate that the perception of four dimensions of justice (i.e., procedural, distributive, interpersonal, and informational) in PA practices positively influences commitment, organizational citizenship behavior (OCB), job satisfaction, intention to stay, and job engagement. As such, organizational justice is a central component in PA. The nurses expect reciprocity in their social relationships in the workplace, especially reciprocity in the psychological aspect of trust, respect, and support [26]. Moreover, Roch and Shanock [37] found that procedural, interactional, interpersonal, and informational justice are associated with social relationships: procedural justice with the organization, and the other three dimensions of justice (i.e., interactional, interpersonal, and informational) with the supervisor. Distributive justice reflects more of an economic exchange relationship (e.g., [37]) that leads us to the next difference:
2. We rarely saw any comments pertaining to distributive justice, which concerns the perceived equity of how benefits and expenses are allocated among group members. Thurston and McNall [22] found distributive justice to be directly related to satisfaction with PA. This idea is typically crucial to the PA process but, surprisingly, none of the nurses in our research brought it up. We believe that the absence of this issue stems from the fact that public sector salary decisions are typically subject to strict regulations and open disclosure, with minimal influence from the PA process. Most of the nurses who mentioned rewards usually referred to a course they wanted the head nurse to approve or recognition in the form of a kind word. This finding converges with the findings of Roch and Shanock [37] that the nurses did not seek an economic exchange related to distributive justice but rather a social exchange that expresses other types of justice. Thurston and McNall [22] found that the four justice dimensions (procedural, distributive, interpersonal, and informational) are distinct (although highly correlated constructs). This result is consistent with our findings of a distinction between dimensions of justice—while the nurses referred mostly to procedural justice and addressed to some extent also interpersonal justice, the other two types of justice were hardly mentioned by them.
3. The nurses did not mention politics as part of the PA process; this result is very plausible because when PAs have administrative purposes such as distributing bonuses, stock options, and other performance-based rewards, there is more politics in the appraisal process whereas when PAs are done for the employee development and feedback purposes, politics decreases [4]. As noted in Section 2, there is less consideration of performance-based rewards in the public hospital and therefore politics occupies less space in the evaluation process.
4. This leads us to the fourth point, which involves the significance of praising hard work and focusing on the individual beyond the role (nursing). Although the PA process is a component of performance management [4], and the nurses want to promote their careers and develop professionally, they also need their contribution to be seen, valued, and acknowledged. This relates directly to Maslow's [38] need hierarchy—the innate need for recognition and appreciation. It once again indicates the importance of appreciation and trust in managerial and organizational sensitivity in the process. Although nurses' PA is not really linked to rewards, it is still very important to them. This finding is connected to the statistics that show that managers (who are the ones who lead the PA process) are a critical factor in the success of organizations and “bad” relationships often lead to the employee turnover [39, 40]. Moreover, while studies among nurses have revealed many pitfalls in the process [7, 25], research among nurses in Israel shows that it has many positive aspects. We have seen many references by the nurses who understand the process, perceived it as valid and reliable and not political, and felt as if they were treated in a courteous, respectful, and dignified manner. We believe that cultural differences have an impact in this case and can explain the difference. In Israel, power distance is one of the lowest in the world [41]. This Israeli culture requires transparent, participatory, and dialog-based processes, in which the nurses feel that they are listened to carefully, decisions are explained to them, and their personal contributions are respected.

The array of themes (see Table 1) indicates the great complexity of the PA process, which is influenced by the characteristics of the appraiser, the characteristics of the appraisee, the characteristics of the PA process (in general), and the characteristics of the feedback session (in particular). While the process is often seen by appraisees as very bureaucratic, forms- and documentation-based, and even technical (a kind of chore), we note the importance of the manager's relationships and interpersonal skills, which express and enable good relationships throughout the process. As such, if we look at the goals of the PA defined in the literature [18, 19, 42], it turns out that the goal of maintaining good relations is seen by the nurses as critical and no less important than development goals or promotion, dismissal, or salary goals, which are administrative goals that the organization often sets for itself in this process. It is possible that the low effectiveness and criticism regarding the PA process derives, inter alia, from the reason that organizational goals do not always fit or correspond with the goals of the process from the employees' perspective (different lenses). Maybe, in the current era, the organizational goal regarding the PA process should move from “hard data” effectiveness to “soft” effectiveness. Namely, improving relationships and attitudes about the process (alongside growth and learning from it) may indirectly increase the organizational desired “hard” effectiveness, and the situation becomes win–win for managers and employees alike. Following Thurston and McNall [22], “If justice perceptions of the procedures and social interactions are important to employees, then these perceptions should influence attitudinal and behavioral reactions beyond the effects of the initial discrepancy between expected and actual performance ratings.” (Thurston and McNall [22], p. 222).

These findings strengthen the research on PA focusing on nonconventional sessions of PAs such as feedforward [43], which are centered on the conversation between manager and employee, the active role of the employee in the review conversation, and the enhancement of strengths, regardless of administrative goals of salary and promotion.

### 4.1. Theoretical and Practical Implications

This research enriches our understanding of nurses' PA process. It offers a more thorough comprehension of the subtleties and complexity of this process in this profession. The study offers valuable insights into nurses' perspectives by capturing their personal experiences and perceptions of PA. This includes identifying obstacles, concerns, and areas for improvement. It can also uncover the hidden factors that influence nurses' performance, such as organizational culture, organizational justice, LMX, and teamwork dynamics. This can help with the creation of assessment systems that better consider their experiences and needs.

Additionally, the study contributes to the development and refinement of theories related to nurse performance and evaluation. For example, the study highlights the importance of the appraisee's characteristics in the PA process, particularly from a SET perspective and the nurses need for social exchange. In addition, the study expands the knowledge regarding PA processes in public and government institutions that are characterized by rigid collective wage agreements rather than individual contracts, and therefore, the relationship between performance and wages is weak.

This study has several important practical implications. First, it can increase the managers' awareness of the importance of the process from the nurses' perspective, not only from the managerial and organizational perspective. For example, if organizational appraisers develop a thorough understanding of employees' perceptions regarding the justice in the PA process, they can adjust their appraisal practices in ways that foster employees' belief that these processes are just.

Moreover, it can provide guidance for creating more useful and effective PA instruments that encompass the entire spectrum of nursing contributions and competencies. Furthermore, it invites interventions regarding soft people skills (human skills) such as supportive communication and care for the one that gives care (the nurses) in the process in general and during the performance session in particular.

Second, it can improve patient health care. Healthcare organizations can create interventions to enhance patient care and safety by better understanding the factors that impact nurse performance.

Third, the study can enhance job satisfaction and retention. This study pinpoints the elements that support increased job satisfaction, employee engagement, and retention among nurses. A more encouraging and helpful work environment for nurses can be established with the help of this information.

Fourth, the findings of this study highlight the importance of creating appraisal processes that are both supportive and development oriented. Healthcare managers should ensure that appraisal sessions provide constructive feedback while fostering an atmosphere of psychological safety, allowing nurses to reflect openly on their professional growth. Providing training for appraisers in communication and feedback delivery can enhance the quality of the sessions and reduce stress and anxiety among appraisees. Policymakers and organizational leaders are encouraged to adopt appraisal frameworks that balance evaluative and developmental aims, aligning performance review systems with the dual goals of accountability and professional development in healthcare settings. This may result in a healthcare system that is more equitable and supportive of nurses as well as patients.

Last, in the PA process, it is recommended to encourage the employees' participation in the feedback session as much as possible. In addition to the fact that employee's initiative in the session encourages his/her future commitment to the (together) set goals, it also allows the appraiser to obtain bottom-up information that is important for improving the performance of the appraiser himself/herself and the organization.

### 4.2. Limitations and Future Research

This study has several limitations. First, the guidance that the nurses received was very general. This was done on purpose to receive the perception of the PA process that came to their minds. However, more specific questions could have given us a more comprehensive perspective on the nurses' views on the PA process. Future study can utilize mixed-method techniques and use more specific questions and some quantifiable metrics to obtain deeper understanding of the nurses' experience. We also suggest a comparative study that compares different PA methods and their impact on organizational outcomes such as employee engagement, job satisfaction, burnout, organizational commitment, and patients' satisfaction.

Future research may also extend the present qualitative findings by adopting more structured quantitative designs, as demonstrated in recent studies [44, 45]. For example, survey-based or experimental approaches could help examine the causal mechanisms linking PA experiences with employee engagement, knowledge sharing, or related organizational outcomes. By combining our qualitative insights with these more formalized designs, future work could provide a more comprehensive understanding of how appraisal processes shape both individual and organizational effectiveness.

Also, the study was carried out in Israel which, as every country, has its own regulations and certification. Therefore, the conclusions drawn from this research may not apply to the larger nursing community due to small sample sizes and particular contexts. We suggest that future research be conducted on various samples from different countries.

Additionally, another limitation concerns the dual role of participants as both students and practicing nurses. Although anonymity and independence from course grading were stressed, it is possible that their position as learners within an academic framework influenced how they articulated their experiences. For instance, some may have sought to present their narratives in ways they perceived as more aligned with academic or evaluative expectations. While we believe the timing of the assignment and the explicit instructions reduced this risk, we nonetheless acknowledge that this dual role may have introduced a subtle bias into the data. In this regard, another limitation is that participants may have drawn on knowledge of PA processes from sources beyond their own experience in the class. As we had no way of fully accounting for this, such prior knowledge could have influenced their narratives.

Also, future studies could extend our approach by asking participants not only to describe aspects of PAs but also to reflect on why they chose to focus on these particular dimensions. Such inquiry may help reveal the underlying meanings and priorities that shape employees' experiences. By linking participants' choices of focus with their interpretations of outcomes, future research may better illuminate how specific characteristics of appraisal systems influence perceptions, engagement, and organizational responses.

Moreover, we suggest that after revising PA processes, future studies should perform a longitudinal study. By tracking changes in nurses' performance and perceptions over time, longitudinal studies can help us better understand the effects of interventions and organizational changes.

## 5. Conclusions

In the current study, we examined the nurses' perspectives on the process of PA they went through. While there are some claims in the literature that the traditional process of PA has become problematic and is not suitable for the dynamic current working world, this research shows otherwise. Nurses care about the PA process, they pay attention to its details, and they hope that their contribution will be positively evaluated. In addition, it is evident that they see the PA process as essential for managing proper relations between supervisors and employees and as a key for procedural justice in organizations in general, and in particular in health organizations, where the relationship between PA and compensation is low.

## Figures and Tables

**Table 1:** Nurses details.

Serial number	Gender	Age	Seniority in current position	Seniority as a nurse	Type of institution/department
1a	Female	38	Over 10 years	Over 10 years	Hospital (children)
1b	Female	37	Over 10 years	Over 10 years	Hospital (psychiatric)
1c	Female	31	3–5 years	3–5 years	Hospital (maternity)
1d	Male	38	5–10 years	5–10 years	Hospital
1e	Male	29	5–10 years	5–10 years	Hospital
1f	Male	37	Over 10 years	Over 10 years	Hospital (recovery)
1g	Female	50	5–10 years	5–10 years	Hospital (dialysis)
1h	Female	41	Under 1 year	Over 10 years	Hospital (internal)
1i	Female	39	1 year–3 years	Over 10 years	Public health
1j	Male	24	1 year–3 years	1 year–3 years	HMO
2a	Female	33	3–5 years	5–10 years	Hospital (maternity)
2b	Female	41	Over 10 years	Over 10 years	Hospital (recovery)
2c	Female	32	3–5 years	3–5 years	Hospital
2d	Female	34	5–10 years	Over 10 years	Hospital (orthopedic)
2e	Male	37	5–10 years	Over 10 years	Hospital (internal)
2f	Female	55	5–10 years	Over 10 years	Hospital (women)
2g	Female	28	3–5 years	3–5 years	Hospital (surgery)
2h	Male	31	5–10 years	5–10 years	Hospital (oncology)
2i	Male	41	1 year–3 years	Over 10 years	Hospital (surgery)
2j	Female	35	1 year–3 years	5–10 years	Hospital (nursing)
3a	Female	40	Over 10 years	Over 10 years	Hospital (maternity)
3b	Female	27	1 year–3 years	1 year–3 years	Hospital (internal)
3c	Female	42	3–5 years	5–10 years	Hospital (ER)
3d	Female	26	1 year–3 years	1 year–3 years	Hospital (surgery)
3e	Female	26	1 year–3 years	1 year–3 years	Hospital (internal)
3f	Female	27	1 year–3 years	1 year–3 years	Hospital
3g	Female	27	5–10 years	5–10 years	Hospital (transplant)
3h	Female	39	Over 10 years	Over 10 years	Hospital
3i	Female	37	Over 10 years	Over 10 years	Hospital
3j	Female	29	3–5 years	3–5 years	Hospital
3k	Female	37	5–10 years	5–10 years	Hospital
3l	Male	48	Over 10 years	Over 10 years	Hospital (ER)
3m	Female	58	Over 10 years	Over 10 years	Hospital (ICU)
3n	Female	24	Over 10 years	Over 10 years	Hospital

**Table 2:** The tree of themes in the research.

3.1.1. Characteristics of the feedback session (the PA interview)	3.1.1.1 The location	
	3.1.1.2 Emotion management in the session	
	3.1.1.3 Improving the appraisal performance	3.1.1.3.1 Setting goals
		3.1.1.3.2 Concerns for improvement or preservation
		3.1.1.3.3 Offering guidance and assistance
	3.1.1.4 Career development	
	3.1.1.5 Bottom-up feedback	

3.1.2. Characteristics of the appraisee	3.1.2.1 Tenure	3.1.2.1.1 Tenure in the organization
		3.1.2.1.2 Tenure in the profession
	3.1.2.2 Attitudes toward the PA process	
	3.1.2.3 Need for appreciation	
	3.1.2.4 Personal context	

3.1.3. Characteristics of the appraiser	3.1.3.1 Appraiser's professionalism regarding the PA	3.1.3.1.1 Strictness
		3.1.3.1.2 Seriousness
	3.1.3.2 Motivation to appraise	
	3.1.3.3 Expressing emotions	

3.1.4. Characteristics of the PA process	3.1.4.1 Frequency and timing	
	3.1.4.2 Justice	3.1.4.2.1 Procedural justice
		3.1.4.2.2 Interactional justice
		Interpersonal justice
		Informational justice
	3.1.4.3 Goals of the process	3.1.4.3.1 Management of career paths
		3.1.4.3.2 Professional guidance
		3.1.4.3.3 Open communication
		3.1.4.3.4 Improvement of the department
	3.1.4.4 Criteria for evaluation	3.1.4.4.1 General assessment
		3.1.4.4.2 Contribution to the organization's success
		3.1.4.4.3 Direct performance
		Performing the tasks
		Meeting objectives
		3.1.4.4.4 Indirect performance
		Initiative behavior
		Relationship with other people
		3.1.4.4.5 Professional knowledge

**Table 3:** Alignment between the thematic coding in the current study, the four dimensions of justice (according to Colquitt [36] and Roch and Shanock [37]), and the perceptions of fair appraisal practices items according to Thurston and McNall [22].

Perceptions of fair appraisal practices items (Thurston and McNall [22])	Justice according to Colquitt [36] and Roch and Shanock [37]	Relevant theme in the current research based on nurses' sample
Assigning raters	Procedural	3.1.3.1, 3.1.3.2, 3.1.4.2.1, 3.1.4.2.2
Setting criteria	Procedural	3.1.1.3, 3.1.3.1.2, 3.1.4.4
Seeking appeals	Procedural	3.1.4.2.2 (signing without agreeing)
Equity decision norm (ratings based on equity)	Distributive	3.1.4.2.1, 3.1.4.2.2
Absence of political goals	Procedural	3.1.4.3
Respect in supervision	Interpersonal	3.1.3.1.2, 3.1.3.2, 3.1.4.2.2
Sensitivity in supervision	Interpersonal	3.1.1.3.3, 3.1.1.4, 3.1.2.4, 3.1.3.3
Clarifying expectations	Informational	3.1.1.3.1, 3.1.1.4
Providing feedback	Informational	3.1.1.3.1, 3.1.1.3.2, 3.1.1.4, 3.1.4.1
Explaining decision	Informational	3.1.3.2, 3.1.4.2.2, 3.1.4.4

## Data Availability

The data that support the findings of this study are available from the corresponding author, upon reasonable request.

## Appendix A

  The Assignment Form (Translated from Hebrew)
  Netanya Academic College
  School of Health Systems Management
  Course Name: Management Skills.
  Assignment: Tell me about the PA process you went through in the organization.
  This is a personal assignment—there is no right or wrong answer.
  It is about your personal story regarding a PA!!!
  Assignment length—at least half a page (you may extend it as needed).
  Submission deadline—by the end of the class on November 24. Submit via the course website submission box.
  After telling your story about the PA you experienced, please answer several short questions, all referring to your situation at the time of the PA you described.
  Mark the most accurate answer!
  Gender: Female; Male.
  Age:
  Seniority in the organization (in years):
  Seniority in the current position:
  Employment framework:
  1. Hospital.
  2. Health services in HMO clinics.
  3. Specialized clinics and institutes.
  4. Other. Specify:
  Field of work:
  1. Medicine.
  2. Nursing.
  3. Pharmacy.
  4. Human Resources.
  5. Procurement.
  6. Maintenance.
  7. Other. Specify:
  Are you responsible for the work of others?
  1. I am not responsible for others' work.
  2. Yes, I am a team or unit leader.
  3. Yes, I am a department head or higher.
  Do you work in a team? Yes; No; Other. Specify:
  The feedback on the performance evaluation you described was conducted as follows:
  1. Face to face.
  2. Remotely via computer (Zoom, etc.)
  3. Other. Specify:
  Note: It is possible that the text will be used completely anonymously for research purposes. If a student objects, please mention it at the end of the assignment. Of course, the grade will not be affected!
